# Uncovering the metabolic-epigenetic links between gene expression and stroke: insights from lactylation pathway MR study

**DOI:** 10.1186/s42466-026-00478-4

**Published:** 2026-03-24

**Authors:** Jiuxu Kan, Yong Hong, Ruoxin Min, Bowen Zhang, Hong Wang

**Affiliations:** Department of Cardiology, The First Affiliated Hospital of Harbin Medical University, Harbin, China

**Keywords:** Ischemic stroke, Lactylation, Mendelian randomization, eQTL analysis, Metabolic regulation

## Abstract

**Background:**

Lactylation, a novel post-translational modification driven by lactate accumulation, has been implicated in neuroinflammation and metabolic stress. However, its causal relevance to ischemic stroke (IS) and its subtypes—large artery stroke (LAS), cardioembolic stroke (CES), and small vessel stroke (SVS)—remains unknown.

**Methods:**

We conducted a two-sample Mendelian randomization (TSMR) analysis to investigate the causal relationships between lactylation-associated gene expression and IS risk. Lactylation-related genes were identified from a recent literature review and intersected with eQTL data from the eQTLGen Consortium (*n* = 31,684). Summary statistics for IS and its subtypes were obtained from large-scale GWAS (total cases = 62,100; controls = 1,234,808). Primary analyses used the inverse-variance weighted (IVW) method, complemented by MR-Egger, weighted median, and sensitivity tests to assess heterogeneity and pleiotropy.

**Results:**

A total of 15 genes and 274 single nucleotide polymorphisms (SNPs) were included. Elevated expression of *SIRT1*, *SMARCA4*, *STMN1*, and *LDHA* was significantly associated with increased risk of IS or its subtypes. In contrast, *SLC16A1*, *SIRT3*, *PFKP*, and *TKT* were inversely associated with stroke risk, suggesting a potential protective role. Most associations were robust across multiple MR models. Pleiotropy and heterogeneity were observed for *SMARCA4* in LAS.

**Conclusion:**

This study provides genetic evidence for the involvement of lactylation-related genes in IS pathogenesis, revealing novel risk-enhancing and protective factors. These findings enhance our understanding of metabolic-epigenetic mechanisms in stroke and suggest potential molecular targets for future interventions.

**Supplementary Information:**

The online version contains supplementary material available at 10.1186/s42466-026-00478-4.

## Introduction

Ischemic stroke (IS) is a leading cause of mortality and long-term disability worldwide, posing a significant burden on aging populations due to its associated neurological and cognitive impairments [1]. According to the TOAST classification, IS comprises three major subtypes with distinct etiologies: large artery stroke (LAS), cardioembolic stroke (CES), and small vessel stroke (SVS) [2]. These subtypes differ substantially in their molecular mechanisms and therapeutic responses. While traditional vascular risk factors such as hypertension and diabetes are well established, the underlying subtype-specific molecular pathways remain incompletely understood. Recently, lactylation, a newly identified post-translational modification that involves covalent binding of lactate to lysine residues, has been implicated in regulating metabolic reprogramming, neuroinflammation, and hypoxic injury [3, 4]. However, its causal relevance to human IS and its subtypes has not yet been clarified.

Lactylation has emerged as a focus of intense investigation due to its multifaceted biological functions. Preclinical studies have shown that lactylation exacerbates ischemic brain injury by modulating ferritinophagy, glycolytic metabolism, and histone modifications [5–7]. For instance, lactylation at lysine 450 of nuclear receptor coactivator 4 (NCOA4) stabilizes the protein, promoting ferroptosis and glycolysis, thereby enlarging infarct size [8]. Similarly, lactylation of ARF1 in astrocytes impairs mitochondrial transfer to neurons and worsens ischemia-reperfusion damage [9]. Clinical studies further report that cerebrospinal fluid (CSF) lactate levels positively correlate with neuroinflammation in IS patients, and lactylated proteins such as *SLC25A4* and *VDAC1* may regulate neuronal apoptosis via calcium signaling pathways [10, 11]. Despite growing mechanistic evidence, the causal relevance of lactylation-related gene expression to IS and its subtypes has not been evaluated using human genetic data.

Although preclinical studies have demonstrated the detrimental roles of lactylation in IS models, critical gaps remain in our understanding of its relevance to human disease. Most existing research is limited to cellular or animal models, lacking validation from large-scale human genetic studies. In particular, the contribution of lactylation-associated gene expression to IS risk, especially across distinct pathological subtypes such as LAS, CES, and SVS, has not been systematically examined. Moreover, genetic variation in key lactylation regulators and their downstream targets has not yet been explored in relation to subtype-specific stroke susceptibility.

This study aims to elucidate the causal effects of lactylation-associated gene expression on IS and its subtypes using a two-sample Mendelian randomization (TSMR) framework [12], providing genetic evidence for the role of lactylation in stroke pathogenesis.

## Methods

### Study design

**Fig. 1:**
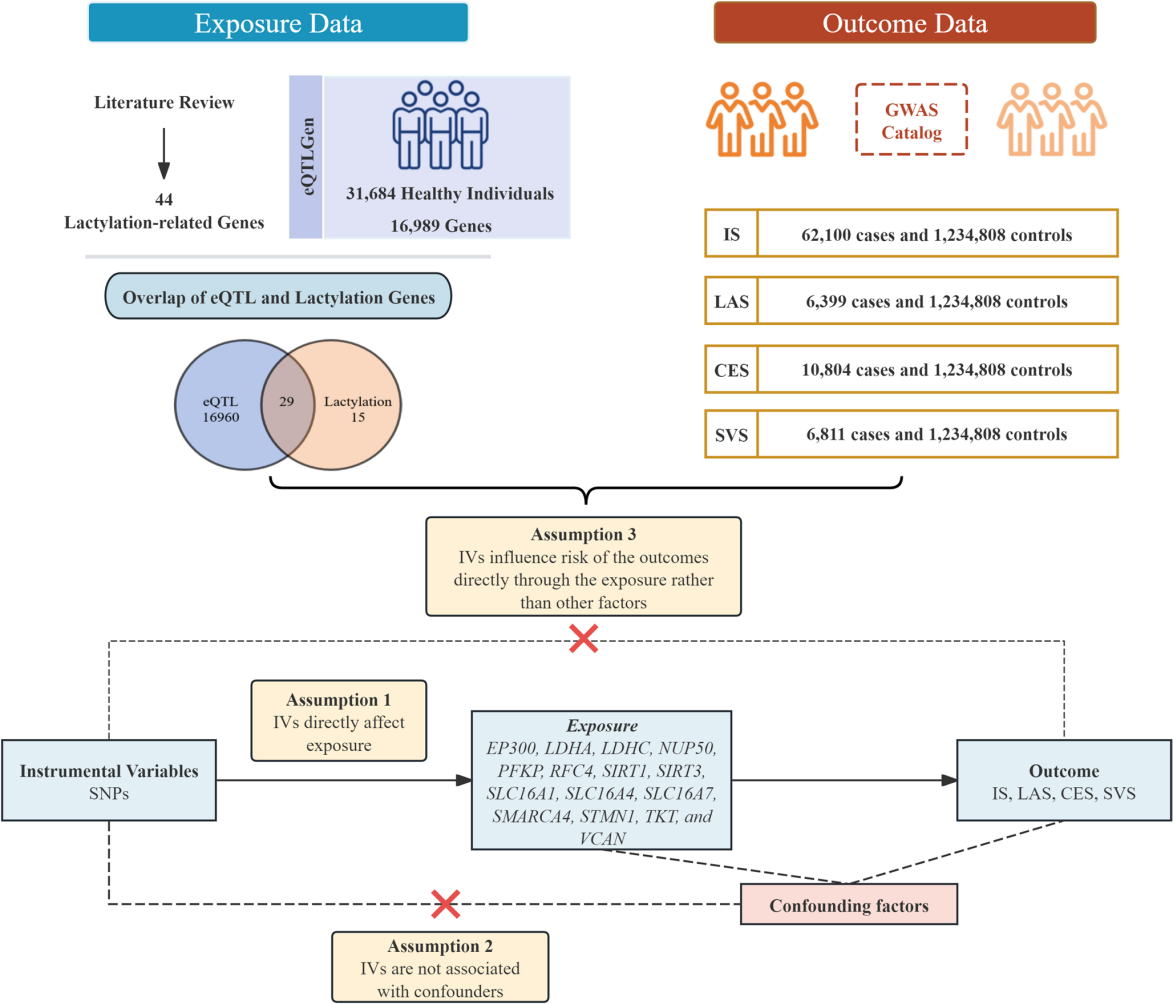
Overview of study design for MR analysis of lactylation-related genes and IS risk. This flowchart illustrates the design of a two-sample MR study evaluating the causal effects of lactylation-associated gene expression on IS and its subtypes. A total of 44 lactylation-related genes were curated through literature review, of which 29 had corresponding cis-eQTLs available in the eQTLGen Consortium dataset (31,684 healthy individuals, 16,989 genes). GWAS summary statistics for IS and its subtypes—including LAS, ES, an SVS—were obtained from the GWAS Catalog, encompassing up to 62,100 cases and 1,234,808 controls of European ancestry. Instrumental variables were selected to satisfy three core MR assumptions: (1) relevance (SNPs are associated with the exposure), (2) independence (SNPs are not related to confounders), and (3) exclusion restriction (SNPs affect the outcome only via the exposure). 15 lactylation-related genes passed the selection process and were included in downstream causal inference analyses

Figure 1 illustrates the overall analysis workflow. Initially, we extracted 44 lactylation-associated genes from a recent comprehensive literature review. Subsequently, we intersected these lactylation-associated genes with expression quantitative trait loci (eQTL) data from the eQTLGen Consortium, deriving instrumental variables (IVs) for MR analysis.

TSMR analyses were then performed for IS and its subtypes (LAS, CES, SVS) from genome-wide association studies (GWAS) summary statistics obtained from the GWAS catalog database. We employed robust quality control and sensitivity analyses to ensure the validity of our findings.

### Exposure data

The eQTL summary statistics used as exposure data were obtained from the eQTLGen Consortium (https://eqtlgen.org/), comprising 31,684 blood samples from healthy European individuals and covering 16,989 genes [13]. After intersecting lactylation-associated genes with eQTLGen data, 29 lactylation-associated genes remained for subsequent analysis (Supplementary Table 1). In this study, cis-eQTLs were defined as genetic variants located within ± 1 Mb of the transcription start site (TSS) of each gene. Only significant cis-eQTLs reported in the eQTLGen dataset were considered as candidate instruments.

### Outcome data

We obtained summary-level GWAS statistics for IS and its clinically relevant subtypes from the GWAS Catalog database [14]. Specifically, the GWAS datasets included IS (GWAS ID: GCST90104540; 62,100 cases and 1,234,808 controls), LAS (GWAS ID: GCST90104542; 6,399 cases and 1,234,808 controls), CES (GWAS ID: GCST90104541; 10,804 cases and 1,234,808 controls), and SVS (GWAS ID: GCST90104543; 6,811 cases and 1,234,808 controls). These large-scale GWAS datasets, derived from populations of European ancestry, provided robust statistical power for MR analyses and facilitated reliable genetic estimates.

### Selection of Genetic Instrumental Variables (IVs)

To ensure the validity and reliability of our TSMR analyses, we implemented stringent quality control criteria for selecting genetic IVs. First, we selected variants demonstrating strong associations with the exposure, retaining only those with F-statistics ≥ 10, calculated as F = β^2^/SE^2^, to minimize weak instrument bias [15]. Subsequently, to ensure independence among instrumental SNPs, we performed linkage disequilibrium (LD) clumping using the European reference panel from the 1000 Genomes Project, retaining only SNPs in weak LD (r² < 0.1) [16]. The relatively lenient LD threshold (r² < 0.1) was chosen to retain sufficient instrumental variants for genes with limited cis-eQTL availability, while minimizing redundancy among correlated SNPs. Furthermore, Steiger filtering was applied to exclude SNPs that explained a larger proportion of variance in the outcome than in the exposure, thereby confirming the correct directionality of the genetic instruments [17]. After applying these rigorous selection criteria, a total of 274 instrumental SNPs corresponding to 15 lactylation-associated genes (*EP300*,* LDHA*,* LDHC*,* NUP50*,* PFKP*,* RFC4*,* SIRT1*,* SIRT3*,* SLC16A1*,* SLC16A4*,* SLC16A7*,* SMARCA4*,* STMN1*,* TKT*,* VCAN*) were retained for subsequent MR analyses (Supplementary Table 1). The strength of these selected instruments was robust, with F-statistics ranging from 30 to 2,901.28, indicating sufficient power to detect potential causal associations. Palindromic SNPs with intermediate allele frequencies were excluded during harmonization to avoid strand ambiguity. Allele harmonization between exposure and outcome datasets was performed using the default settings of the “TwoSampleMR” (version 0.5.7) package to ensure alignment of effect alleles.

### MR analyses

We conducted TSMR analyses using the R package “TwoSampleMR”. The inverse-variance weighted (IVW) method was used as the primary statistical method, providing optimal statistical power under the assumption of no horizontal pleiotropy [18]. Additionally, we employed complementary MR methods, including MR-Egger [19], weighted median [20], weighted mode, and simple mode, to validate the robustness of our findings.

### Sensitivity analyses

To verify the robustness of our MR findings and assess potential biases arising from heterogeneity and horizontal pleiotropy, multiple sensitivity analyses were systematically conducted. First, we performed the MR-Egger intercept test to evaluate the presence of horizontal pleiotropy [21], where a statistically significant intercept (*P* < 0.05) indicates potential pleiotropy among genetic instruments. Next, we applied Cochran’s Q test to assess heterogeneity across the SNPs included as instruments [22], with a significant Q statistic (*P* < 0.05) suggesting notable heterogeneity. Additionally, we utilized the MR-PRESSO global test to detect and correct for potential outlier SNPs that might bias the causal estimates [23]. Finally, a leave-one-out analysis was conducted to assess whether any single SNP disproportionately influenced the overall MR results, further confirming the stability and reliability of causal estimates [24]. These comprehensive sensitivity analyses were critical to ensure the validity and interpretability of the observed causal associations.

## Results

### Selection of genetic IVs

Following stringent quality control steps, we identified 274 valid genetic IVs representing 15 lactylation-associated genes (*EP300*,* LDHA*,* LDHC*,* NUP50*,* PFKP*,* RFC4*,* SIRT1*,* SIRT3*,* SLC16A1*,* SLC16A4*,* SLC16A7*,* SMARCA4*,* STMN1*,* TKT*, and *VCAN*) (Supplementary Table 1). The strength of these instrumental SNPs was robust, with F-statistics ranging from 30 to 2,901.28, indicating sufficient power to detect causal associations reliably.

### MR analyses between lactylation-associated genes and IS

**Fig. 2:**
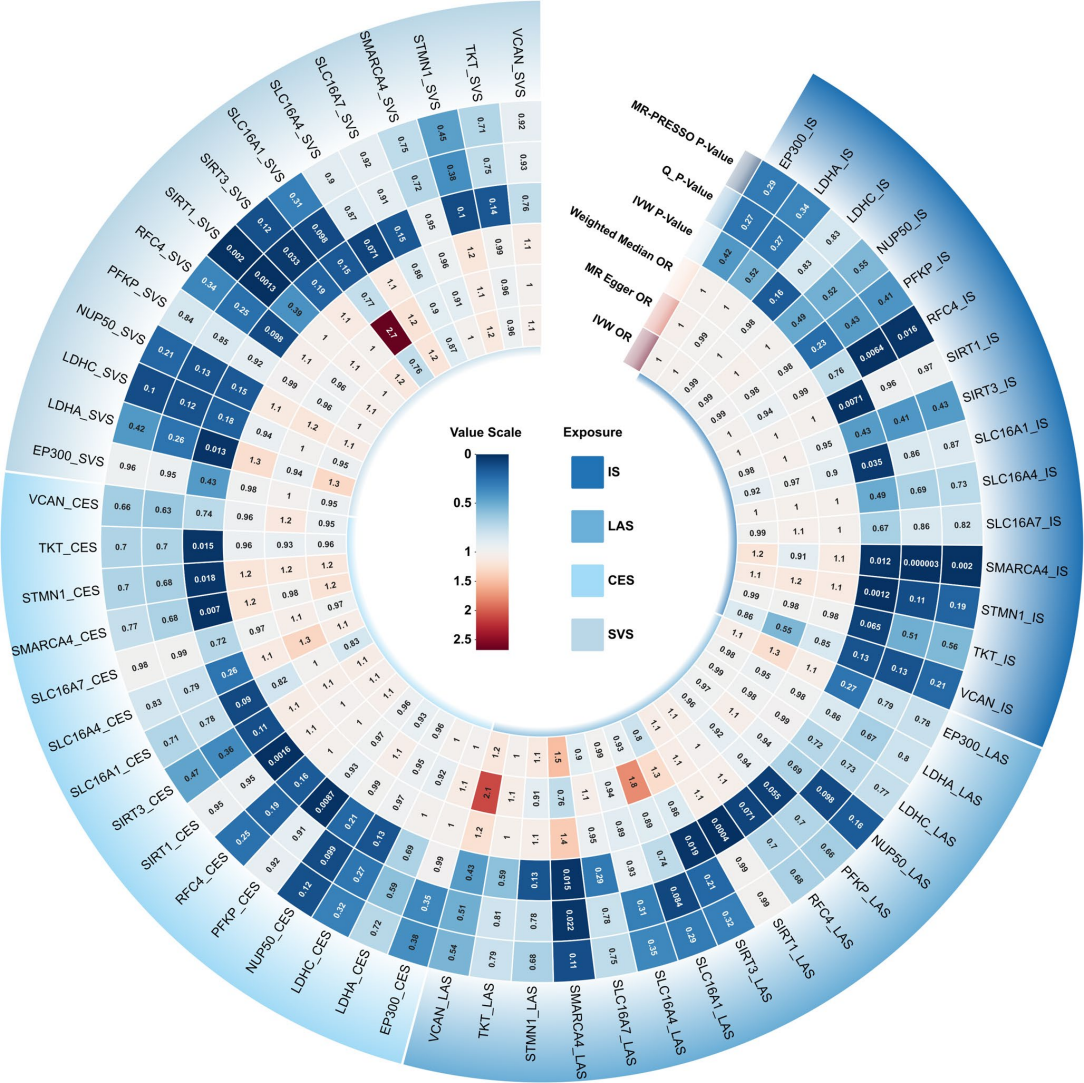
Circular heatmap of MR estimates for the associations between lactylation-related gene expression and IS and its subtypes. This circular heatmap displays MR results for 15 lactylation-related genes across four stroke phenotypes: IS, LAS CES, and SVS. From inner to outer rings, the following metrics are shown: IVW OR, MR-Egger OR, weighted median OR, IVW P-values, Cochran’s Q P-values, and MR-PRESSO global test P-values. The value scale indicates the magnitude of odds ratios (centered at 1.0), with red representing increased risk and blue representing decreased risk. Significant IVW P-values (*P* < 0.05) are bolded, highlighting key gene-stroke associations. Gene-outcome pairs are arranged clockwise along the outermost circle, allowing visualization of subtype-specific effects

TSMR analyses were conducted to assess causal associations between genetically predicted gene expression levels of lactylation-associated genes and IS risk and its subtypes (LAS, CES, and SVS) (Fig. 2).

**Fig. 3:**
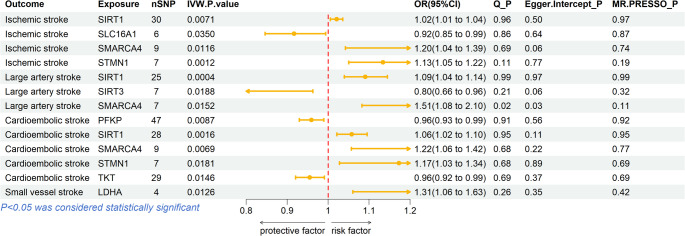
Forest plot of MR results for lactylation-related genes associated with IS and its subtypes. This forest plot summarizes significant associations between genetically predicted expression of lactylation-related genes and the risk of IS, LAS CES, and SV. Displayed are the number of SNPs used (nSNP), IVW P-values, odds ratios with 95% CI], Cochran’s Q P-values (Q_P), MR-Egger intercept P-values (Egger.Intercept_P), and MR-PRESSO global test P-values (MR.PRESSO_P). Horizontal bars represent the 95% CI for each causal estimate, with the dashed vertical red line indicating the null effect (OR = 1). Estimates to the left of the line indicate protective effects, while those to the right indicate increased risk. P-values < 0.05 were considered statistically significant and are highlighted in bold

**Fig. 4:**
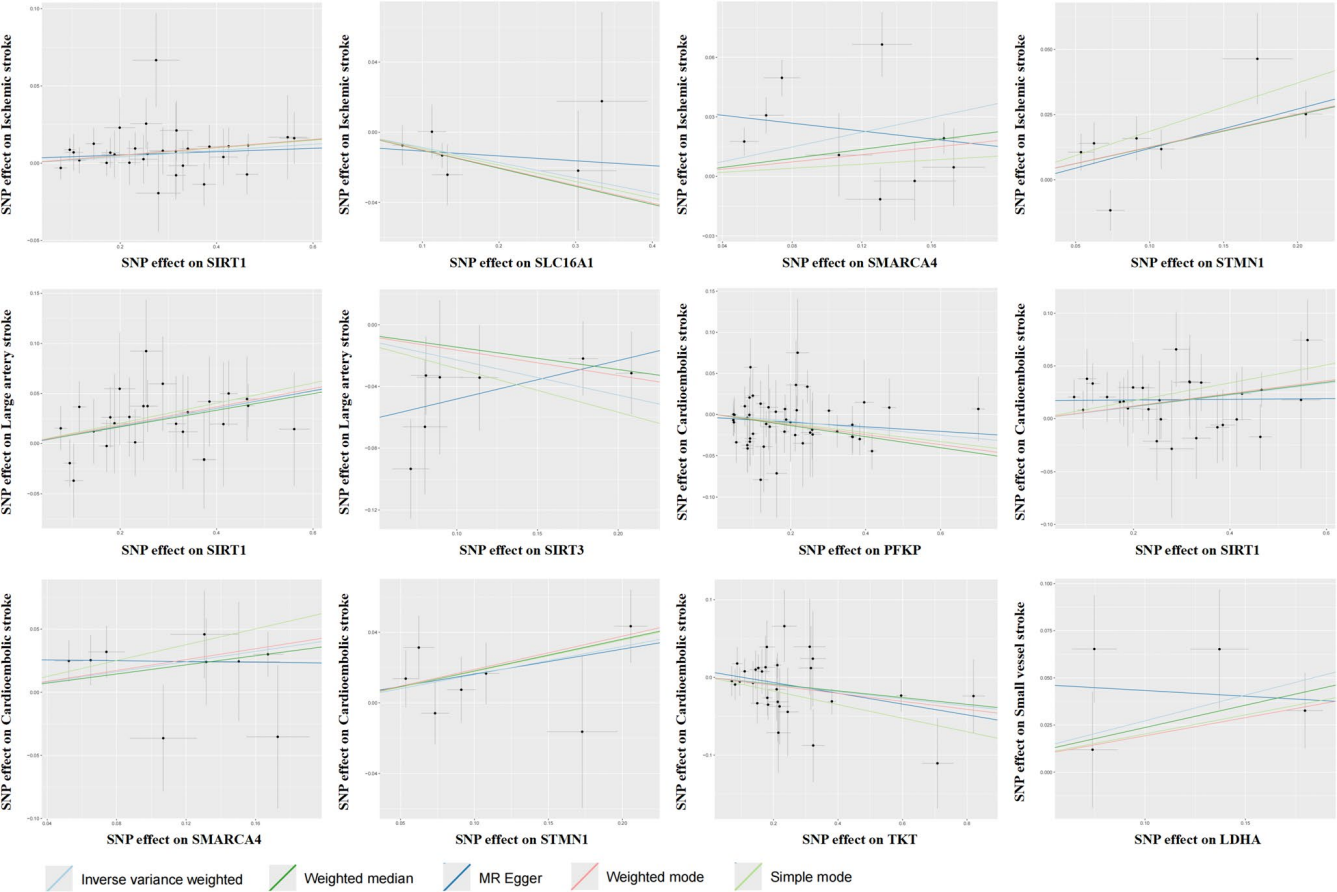
Scatter plots of MR estimates for lactylation-related genes significantly associated with IS and its subtypes. Each panel displays the relationship between SNP-exposure and SNP-outcome effects for one gene-stroke pair identified as statistically significant in the primary MR analysis. The x-axis represents the effect of each SNP on the expression level of a lactylation-related gene, while the y-axis shows the corresponding SNP effect on IS or its subtypes: LAS CES, and SV. Colored lines represent causal estimates derived from different MR methods: IVW (light blue), MR-Egger (dark blue), weighted median (green), weighted mode (red), and simple mode (light green). Consistency in the direction of these lines supports the robustness of the observed associations. Each dot represents an individual SNP used in the analysis, and the vertical/horizontal bars indicate standard errors of SNP effects on the outcome and exposure, respectively

In the primary IVW MR analyses, the expression levels of four lactylation-associated genes displayed significant causal associations with IS risk (*P* < 0.05) (Supplementary Table 2, Figs. 3 and 4). Specifically, increased expression of *SIRT1* (OR = 1.02, 95% CI: 1.01–1.04; *P* = 0.0071), *SMARCA4* (OR = 1.20, 95% CI: 1.04–1.39; *P* = 0.0116), and *STMN1* (OR = 1.13, 95% CI: 1.05–1.21; *P* = 0.0012) were associated with a higher risk of IS, whereas increased expression of *SLC16A1* (OR = 0.92, 95% CI: 0.85–0.99; *P* = 0.0350) was associated with a lower risk.

Further analyses of IS subtypes revealed subtype-specific associations. For LAS (Supplementary Table 3, Figs. 3 and 4), higher expression of *SIRT1* (OR = 1.09, 95% CI: 1.04–1.15; *P* = 0.0004) and *SMARCA4* (OR = 1.51, 95% CI: 1.09–2.08; *P* = 0.0152) increased the risk, whereas elevated *SIRT3* expression was protective (OR = 0.80, 95% CI: 0.66–0.97; *P* = 0.0188). For CES (Supplementary Table 4, Figs. 3 and 4), higher expression of *PFKP* (OR = 0.96, 95% CI: 0.93–0.99; *P* = 0.0087) and *TKT* (OR = 0.96, 95% CI: 0.93–0.99; *P* = 0.0146) reduced the risk, while increased expression of *SIRT1* (OR = 1.06, 95% CI: 1.02–1.10; *P* = 0.0016), *SMARCA4* (OR = 1.22, 95% CI: 1.06–1.41; *P* = 0.0069), and *STMN1* (OR = 1.17, 95% CI: 1.03–1.33; *P* = 0.0181) were associated with increased risk. For SVS (Supplementary Table 5, Figs. 3 and 4), elevated *LDHA* expression significantly increased stroke risk (OR = 1.31, 95% CI: 1.06–1.62; *P* = 0.0126).

### Sensitivity analyses

**Fig. 5:**
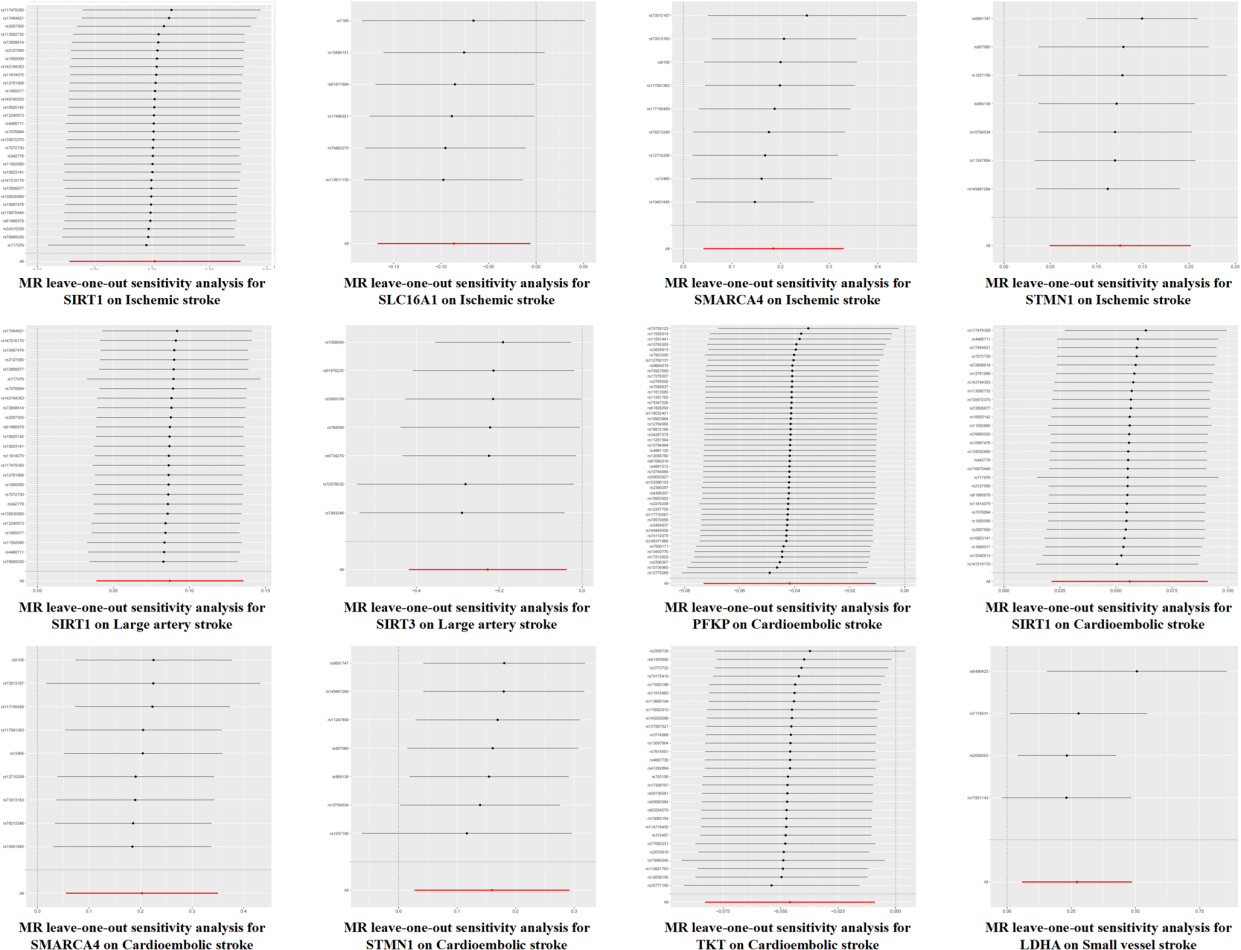
Leave-one-out sensitivity analyses for associations between lactylation-related genes and IS outcomes. Each panel displays a leave-one-out analysis evaluating the robustness of MR estimates for gene-stroke associations. In these analyses, each individual SNP is sequentially removed to assess its influence on the overall causal estimate. The x-axis represents the MR effect size after excluding each SNP, and the y-axis denotes the corresponding SNP identifier. The horizontal lines represent 95% confidence intervals for the effect estimates. Results showing minimal variation across all iterations indicate that no single SNP disproportionately influenced the causal inference

Multiple sensitivity analyses confirmed the robustness of our MR findings. The MR-Egger intercept tests indicated no significant horizontal pleiotropy for the majority of genes (all Egger lntercept *P* > 0.05), except for *SMARCA4* in LAS (Egger lntercept *P* = 0.03) (Supplementary Tables 3 and Fig. 3), suggesting potential pleiotropy influencing this specific association. Cochran’s Q tests revealed minimal heterogeneity among SNP instruments for most associations (Q statistic *P* > 0.05), except for *SMARCA4* in LAS (Q statistic *P* = 0.02) (Supplementary Tables 3 and Fig. 3). However, MR-PRESSO analysis indicated no influential outliers significantly altering the causal estimates across all significant associations. Moreover, leave-one-out analyses demonstrated that no single SNP disproportionately influenced the observed relationships, further supporting the stability and reliability of the results presented (Fig. 5).

## Discussion

In this study, we systematically investigated the causal relationships between genetically predicted expression levels of lactylation-associated genes and IS risk using two-sample MR. Our primary findings revealed significant causal associations between increased expression of several lactylation-related genes, notably *SIRT1*, *SMARCA4*, and STMN1, and an elevated risk of IS. Conversely, elevated expression levels of specific lactylation-associated genes, including *SLC16A1*,* SIRT3*,* PFKP*, and *TKT*, were identified as protective factors, conferring decreased susceptibility to IS or its clinical subtypes such as LAS and CES. Taken together, these findings provide robust genetic evidence supporting the important causal roles of lactylation-related biological pathways in the pathogenesis of IS, highlighting gene-specific and subtype-specific effects that merit further mechanistic exploration and validation. Recent work has highlighted the importance of thrombo-inflammatory mechanisms during ischemia–reperfusion injury in both experimental and human stroke, further emphasizing the complex interaction between metabolic stress, inflammation, and vascular pathology in stroke pathogenesis [25].

*SIRT1* (silent information regulator 2 homolog 1), a NAD⁺-dependent deacetylase, has been widely studied for its regulatory roles in energy metabolism, oxidative stress, neuroinflammation, and neuronal survival. Experimental studies have shown that *SIRT1* exerts neuroprotective effects in IS models by modulating critical signaling pathways, such as PGC-1α, FOXO, p53, HMGB1, and NF-κB [26, 27]. Specifically, *SIRT1* deacetylation activity suppresses NLRP3 inflammasome activation and inflammatory cytokine production, promotes mitochondrial biogenesis, and enhances neuronal antioxidant defenses [28]. Activation of *SIRT1* has been demonstrated to reduce infarct volume and improve neurological recovery in animal models of IS [29]. Moreover, *SIRT1* modulates microglial polarization from the pro-inflammatory M1 phenotype to the anti-inflammatory M2 phenotype, thereby reducing neuroinflammation and supporting post-stroke repair [30, 31]. Despite these protective mechanisms, our MR analysis revealed that genetically predicted higher expression of *SIRT1* was causally associated with an increased risk of IS and its subtypes, particularly large artery and CES. This finding appears to contradict prior experimental evidence but can be explained through several important considerations. First, the eQTL data used in our analysis were derived from peripheral blood, which may not fully reflect *SIRT1* expression and function in the central nervous system. *SIRT1* expression in peripheral tissues may represent a compensatory response to systemic inflammation or metabolic stress, conditions that are themselves risk factors for stroke [32]. Second, MR estimates capture the effects of lifelong genetically determined expression levels, which differ fundamentally from acute or transient upregulation observed in most preclinical models. Chronic overexpression of *SIRT1* might lead to maladaptive metabolic or immune activation over time, thereby promoting vascular pathology. In addition, *SIRT1* activity is highly context-dependent. Although it can suppress inflammation in certain scenarios, sustained *SIRT1* activation has also been shown to enhance glycolytic flux and lactate production, contributing to increased lactylation and metabolic stress under ischemic conditions [33]. Furthermore, genetic instruments that regulate *SIRT1* expression may exert pleiotropic effects on other stroke-related pathways, such as endothelial dysfunction or oxidative damage, even if statistical evidence for horizontal pleiotropy was not detected in our sensitivity analyses. Taken together, while *SIRT1* has demonstrated neuroprotective effects in experimental stroke models, our findings highlight the complex and potentially dual roles of this regulator in stroke pathogenesis. These results underscore the need for tissue-specific and temporal investigations into *SIRT1* biology, as well as functional validation of genetically identified risk pathways.

While there is no direct experimental proof connecting *SMARCA4* and *STMN1* to IS, our MR analysis suggests these genes as risk factors, indicating possible but not yet fully explored roles in stroke development. *SMARCA4* encodes a core ATPase subunit of the SWI/SNF chromatin remodeling complex and is known to regulate transcriptional programs involved in inflammation, vascular remodeling, and blood–brain barrier integrity [34, 35]. Dysregulated *SMARCA4* expression may exacerbate ischemic injury by promoting maladaptive gene expression in endothelial or glial cells. *STMN1* encodes stathmin, a microtubule-destabilizing protein critical for neuronal cytoskeletal dynamics [36]. Elevated expression of *STMN1* has been associated with axonal damage and impaired synaptic function in models of neurodegeneration [36, 37], suggesting that its overactivity during ischemia may disrupt neuronal recovery through cytoskeletal instability. Our findings introduce *SMARCA4* and *STMN1* as novel genetic contributors to stroke susceptibility. Although the precise mechanisms remain to be elucidated, these results provide a rationale for further investigation using brain-specific transcriptomic data and functional studies to clarify their roles in cerebrovascular injury.

The MR analysis revealed that *LDHA* (lactate dehydrogenase A) is a gene that raises the risk of SVS, suggesting it may play a pathogenic role via metabolic and neuroinflammatory mechanisms. *LDHA* is a key glycolytic enzyme that catalyzes the conversion of pyruvate to lactate, thereby driving anaerobic metabolism and promoting lactate accumulation during ischemic conditions. In cerebral ischemia/reperfusion (I/R) injury models, *LDHA* expression is significantly upregulated in association with HIF-1α, contributing to the pro-inflammatory activation of microglia. Inhibiting the HIF-1α/LDHA axis has been shown to reduce the release of pro-inflammatory cytokines, attenuate neuronal death, and improve neurological recovery, highlighting the detrimental role of *LDHA* in stroke pathophysiology [38]. Beyond microglia, *LDHA* also contributes to astrocytic metabolic dysregulation during ischemic stress. In oxygen-glucose deprivation/reoxygenation (OGD/R) models, suppression of the Wnt/β-catenin pathway downregulates glycolytic enzymes including *LDHA*, leading to impaired glucose metabolism, ferroptosis, and exacerbated brain injury [39]. Moreover, lactate produced by *LDHA* enhances lysine lactylation (Kla) of neuronal proteins, particularly during acute ischemia. This post-translational modification activates A1-type neurotoxic astrocytes and worsens cerebral infarction. Pharmacological inhibition of *LDHA* significantly reduces lactate accumulation and infarct volume, suggesting therapeutic potential [40]. *LDHA* also appears to participate in epigenetic regulation following ischemia. Ischemia-induced lactate buildup promotes histone H3K9 lactylation (H3K9la), which in turn transcriptionally upregulates *LDHA* and HIF-1α, establishing a feed-forward loop that drives sustained glycolysis and inflammation. Notably, overexpression of *SMEK1* in microglia suppresses *LDHA* expression and improves neurological outcomes, while SMEK1 deficiency results in excessive lactate generation and worsened injury via the PDK3-PDH pathway [7]. In addition, non-coding RNAs such as miR-19a-3p and miR-143 have been shown to regulate *LDHA* expression post-stroke. These microRNAs inhibit glycolytic flux and reduce cell apoptosis by targeting *LDHA* and associated enzymes such as HK2 and PKM2. Glycine supplementation was found to reverse miR-19a-3p-mediated *LDHA* suppression, thereby restoring metabolic balance and promoting cell survival [41–43]. Taken together, our findings support a causal link between increased *LDHA* expression and heightened stroke risk, particularly in SVS. These results are in line with existing evidence that *LDHA*-driven lactate accumulation exacerbates ischemic brain injury via metabolic dysregulation, protein lactylation, neuroinflammation, and epigenetic remodeling. Targeting *LDHA*—whether via genetic regulation, enzyme inhibition, or dietary compounds such as naringenin (NAR)—may represent a promising therapeutic approach to mitigate SVS burden [40, 44].

*SIRT3* was identified as a protective factor against LAS in our MR analysis, supporting its known roles in mitochondrial homeostasis, oxidative stress reduction, and neurovascular protection. *SIRT3*, a mitochondrial NAD⁺-dependent deacetylase, is critically involved in the cellular response to ischemic injury by preserving mitochondrial function and limiting neuroinflammation. Experimental studies have demonstrated that *SIRT3* knockout mice exhibit worsened neurological outcomes following IS, including impaired neurogenesis and angiogenesis, alongside downregulation of *VEGF*, *AKT*, and *ERK* signaling [45]. Mechanistically, *SIRT3* suppresses HIF-1α signaling to regulate *VEGF* expression, thereby protecting the blood–brain barrier (BBB) and attenuating inflammatory injury [46]. In ischemic conditions, loss of *SIRT3* promotes the opening of mitochondrial permeability transition pores (mPTPs) via increased expression of *VDAC1* and *ANT1*, which in turn accelerates neuronal apoptosis. Conversely, overexpression of *SIRT3* reduces caspase-3 activation and preserves mitochondrial integrity [47]. Furthermore, *SIRT3* activates mitophagy via the AMPK pathway and the PINK1/Parkin axis, enhancing mitochondrial turnover and reducing apoptosis in ischemic neurons (e.g., through downregulation of Bax and caspase-3) [48]. In endothelial cells, *SIRT3* restoration after oxygen-glucose deprivation/reoxygenation (OGD/R) reduces ROS generation and inhibits apoptosis, and agents such as dl-3-n-butylphthalide (NBP) exert their protective effects by upregulating *SIRT3* [49]. *SIRT3* also modulates post-stroke inflammation. Its deficiency enhances NLRP3 inflammasome activation and the release of IL-1β and IL-18, while impairing neural stem/progenitor cell (NSPC) proliferation by downregulating Nestin and Sox2 expression [50]. Pharmacological inhibition of NLRP3 reverses these effects, supporting SIRT3’s anti-inflammatory role. In microglia, SIRT3 promotes migration to ischemic regions via the CX3CR1-G protein signaling axis, facilitating clearance of damaged tissue [51]. At the vascular level, *SIRT3* improves tight junction protein expression (occludin, ZO-1) through the PPAR-γ/p38 MAPK pathway, contributing to BBB stabilization [52]. In parallel, it activates the mitochondrial unfolded protein response (UPRmt) via the FoxO3/Sphk1 pathway, maintaining mitochondrial membrane potential and reducing ROS accumulation, thereby mitigating ischemia/reperfusion injury [53]. Finally, natural compounds such as stilbene glycoside and active fraction of Polyrhachis vicina have been shown to enhance *SIRT3* expression, promoting mitophagy and angiogenesis, and improving neurological recovery after stroke [54]. Collectively, these findings provide strong biological plausibility for our MR results and support *SIRT3* as a key neuroprotective factor in IS, particularly within large-vessel pathology.

Although direct evidence linking *SLC16A1*,* PFKP*, and *TKT* to IS is lacking, our MR findings suggest these genes may exert protective effects via metabolic regulation. *SLC16A1* encodes *MCT1*, a lactate transporter critical for maintaining metabolic balance during ischemia [55]. Its upregulation may enhance lactate clearance, reduce acidosis, and limit harmful lactylation [56]. *PFKP*, a key glycolytic enzyme, may sustain ATP production under hypoxic conditions, supporting neuronal survival [57]. *TKT*, part of the pentose phosphate pathway, contributes to NADPH generation and antioxidant defense [58]. Elevated *TKT* expression could help counteract oxidative stress and support vascular integrity [58]. These genes highlight the importance of energy metabolism and redox homeostasis in stroke protection. Our study provides genetic evidence linking their expression to reduced stroke risk and encourages further mechanistic exploration.

This study provides genetic evidence supporting causal associations between lactylation-related gene expression and IS risk, revealing both risk-enhancing and protective metabolic regulators across stroke subtypes. However, this study has several limitations that should be acknowledged. First, the eQTL data were derived from blood samples, which may not fully reflect gene expression profiles in brain tissues. Although blood-based eQTLs have been shown to correlate with some brain-specific regulatory mechanisms, future studies using brain-derived or tissue-specific transcriptomic data may provide more accurate insights. Second, all GWAS and eQTL datasets used in this study were based on individuals of European ancestry, which may limit the generalizability of our findings to other populations. Third, although sensitivity analyses supported the robustness of most results, potential horizontal pleiotropy and heterogeneity were observed for *SMARCA4* in LAS, suggesting this association should be interpreted with caution.

Future research should focus on validating these findings in independent populations and integrating single-cell transcriptomics or brain-specific eQTL databases. In addition, experimental studies using gene editing or animal models are warranted to explore the mechanistic roles of lactylation-related genes in IS pathogenesis and to assess their potential as therapeutic targets.

### Conclusion

In summary, this study leveraged large-scale eQTL and GWAS datasets to explore the causal relationships between lactylation-associated gene expression and IS, including its major subtypes. Using TSMR, we identified several genes—such as *SIRT1*, *SMARCA4*,* STMN1*, and *LDHA*—that may increase stroke susceptibility, while others, including *SLC16A1*,* SIRT3*,* PFKP*, and *TKT*, were associated with reduced risk. These findings provide new insights into the metabolic and epigenetic mechanisms underlying stroke pathogenesis and highlight gene-specific and subtype-specific effects. Future experimental and transcriptomic studies are warranted to validate these associations and to investigate their potential as therapeutic targets for personalized stroke prevention and intervention.

## Supplementary Information

Supplementary Material 1

Supplementary Material 2

Supplementary Material 3

Supplementary Material 4

Supplementary Material 5

## Data Availability

All data used in this study were obtained from publicly available resources. eQTL summary statistics were derived from the eQTLGen Consortium (https://www.eqtlgen.org), comprising 31,684 whole blood samples from individuals of European ancestry. Summary-level genome-wide association study (GWAS) data for IS and its subtypes were obtained from the GWAS Catalog (https://www.ebi.ac.uk/gwas/), under accession numbers GCST90104540 (IS), GCST90104542 (LAS), GCST90104541 (CES), and GCST90104543 (SVS). All datasets are freely accessible and were used in accordance with the data usage policies of their respective sources.
